# Development and refinement of patient care recommendations in brachytherapy for locally advanced cervical cancer using nominal group technique workshops

**DOI:** 10.1007/s00520-024-08997-z

**Published:** 2024-12-13

**Authors:** Pauline Humphrey, Emma Dures, Peter Hoskin, Jenny Johnston, Louise Reardon, Fiona Cramp

**Affiliations:** 1 College of Health, Science & Society, University of the West of England, Bristol, UK; 2 Bristol Cancer Institute, University Hospitals Bristol and Weston NHS Foundation Trust, Bristol, UK; 3 Division of Cancer Sciences, University of Manchester, Manchester, UK; 4 Mount Vernon Cancer Centre, Northwood, UK

**Keywords:** Nominal group technique, Workshops, Locally advanced cervical cancer, Patient care, Recommendations

## Abstract

**Purpose:**

Patient experiences of brachytherapy for locally advanced cervical cancer (LACC) are widely variable, with reports of difficult and traumatic experiences and aspects of care requiring improvement. The aim of this study was to develop patient care recommendations and consult with key stakeholders to review, refine and prioritise recommendations.

**Methods:**

Phase 1: Patient care recommendations were developed from qualitative exploratory study data. Phase 2: Service users and providers with recent experience of brachytherapy for LACC were recruited to online nominal group technique (NGT) workshops. Four NGT stages were followed: (1) initial voting and silent generation; (2) round robin; (3) clarification; and (4) prioritisation. Voting data were summed across the workshops, deriving inter-group scores. Qualitative data were analysed through content analysis.

**Results:**

Phase 1: Fifty-one patient care recommendations were developed. Phase 2: Thirteen participants took part in three online NGT workshops, with a combination of service users and providers. Initial recommendations were voted on; four new recommendations were added; minor changes were made and second voting was undertaken. Recommendations were positively received with 25 recommendations scoring maximum points from all participants. An importance score above 90% was given to 46 recommendations. The remaining recommendations received scores between 74 and 90%.

**Conclusions:**

NGT workshops facilitated collaboration between key stakeholders, discussing, refining and prioritising patient care recommendations, leading to verification of achievable and relevant recommendations. These provide a foundation for future development of guidelines and subsequent implementation into clinical practice, aiming to improve consistency of care and patient experiences of brachytherapy for LACC.

## Introduction

Brachytherapy for locally advanced cervical cancer (LACC) is a type of internal radiotherapy where a radioactive source is introduced into the vagina, cervix and uterus through hollow applicators [1]. Over the past 20 years, developments in imaging, computer planning and applicator design have led to delivery of higher tumour dose and lower dose to surrounding sensitive structures [2, 3]. The purpose of these developments has been to improve local tumour control and reduce treatment related side effects. Technological developments have increased the complexity of the brachytherapy planning stages, typically leading to an increase in overall time with applicators in-situ [4]. A UK survey reported wide variation in time with applicators in place, ranging from 3 to 52 h, and 11 different treatment regimens, with some regimens include 1 or 2 overnight stays with applicators in place and some with 3 or 4 day case procedures [5].

Brachytherapy for gynaecological cancer is an invasive treatment, with reports of subsequent post-traumatic stress disorder [6]. A systematic literature review found that brachytherapy for gynaecological cancer can cause pain, anxiety and distress [7]. A programme of research was carried out to explore patient experiences of brachytherapy and their ideas for how treatment can be improved and distress reduced. Patient interviews with 35 participants recruited from 4 UK centres showed widely variable experiences of brachytherapy, with some participants reporting difficult and traumatic experiences, periods of uncontrolled pain and perceptions of poor care. However, some participants were more positive, reporting little or no pain and overall good experiences of care. Participants also gave their views on ways that brachytherapy experiences could be improved for future patients [8].

The aim of this study was to develop patient care recommendations and consult with key stakeholders to review, refine and prioritise recommendations. The objectives were (1) to use the patient interview data [8] and UK survey data [5] to develop a list of patient care recommendations; (2) consult with key stakeholders in NGT workshops to review usefulness, potential feasibility and relevance of the recommendations; and (3) refine and prioritise the recommendations.

## Methods

This study was carried out in two phases:

### Phase 1: Drawing up a list of potential patient care recommendations

In an iterative process, informed by the research literature, the first author (PHu) used previous survey [5] and interview data [8] to develop an initial list of patient care recommendations. Using field notes and summaries of the 35 patient interviews, free text comments from the UK survey, all data highlighting areas requiring improvement were systematically categorised. These findings were tabulated to display positive and negative experiences; helpful and unhelpful interventions; explicit suggestions for improvements and implied improvements inferred from interview participants’ narratives. An audit trail of this development process was shared and discussed with the research steering group (PhD supervisors and co-authors ED, PHo and FC; patient research partners and co-authors LR and JJ). This enabled conversion of table contents to a potential list of patient care recommendations, grouped under topic headings, in a format suitable for workshop participant voting.

### Phase 2: Nominal group technique (NGT) workshops

A co-design approach was chosen as this is well suited to health services research, benefitting from the knowledge and experience of patients to enhance implementation and optimise impact [9–11]. NGT, a mixed method approach using qualitative techniques to obtain quantitative results [12], was selected for workshops with key stakeholders. NGT involves expression of individual opinions within a group setting, therefore known as a ‘nominal’ group technique. It is a consensus method used to develop clinical guidelines in evidence-based healthcare [11, 13], allowing for free exchange of opinions and generation of ideas within a structured and non-hierarchical discussion forum [12, 14–17]. Key stakeholders identified were brachytherapy service users (patients) and service providers (healthcare providers).

Ethical approval was obtained from the University of the West of England Health and Applied Sciences Faculty Research Ethics Committee (REF: HAS.21.10.020), and the study was conducted in accordance with the principles of the Declaration of Helsinki.

Purposive recruitment was undertaken to include individuals with brachytherapy experience from a patient or healthcare professional perspective. Service providers were eligible for study inclusion if they had experience of working in brachytherapy for LACC in a UK setting during the previous 5 years, including ward and theatre nurses, clinical nurse specialists, radiographers, oncologists, anaesthetists, anaesthetic assistants and clinical psychologists. Service users were eligible for participation if they had experienced brachytherapy for LACC in a UK hospital in the previous 5 years, were over 18 years old, able to communicate verbally in English, able to complete the online Qualtrics survey to confirm eligibility and online consent (hyperlink sent via email) and access Zoom (video and audio) in a private space and had capacity to consent to take part in the study.

Healthcare professionals were invited to take part in the study through professional contacts, for example through the UK Brachytherapy Radiographers Forum (a Society and College of Radiographers special interest group). Service users were recruited through social media. National charities such as ‘Jo’s Cervical Cancer Trust’ and The Pelvic Radiation Disease Association (PRDA) emailed study information to their contacts who had expressed interest in radiotherapy related research. Twitter (now known as X) was used to advertise the research, using tags to alert some cancer charities to the tweet. Service users and service providers with an interest in the research contacted PHu by email.

To provide a balance of service providers and service users for a manageable online workshop, the team planned for up to 8 members per workshop with up to 4 sequential workshops. This was considered sufficient to allow a meaningful analysis of voting or ranking data, not aiming for statistical representativeness but an attempt to include participants with a range of views and experiences of brachytherapy [18, 19]. The final number of participants and workshops was determined by the number of potential participants completing the online Qualtrics questionnaire, availability to attend a workshop at the same time as other participants and a combination of service users and service providers from different professions and different centres so that workshops were informed by a range of perspectives. Care was taken to avoid service users and service providers from the same centre participating in the same workshop.

A script for the NGT workshop was developed, including ground rules and workshop schedule.

NGT workshops were conducted using Zoom. In advance of the meetings, participants were sent an email outlining the NGT process, ground rules around confidentiality, respect and protection of participants’ identity. The email included the potential patient care recommendations developed in Phase 1 and explanatory notes. Participants were asked to read and make notes of suggestions for wording amendments or new recommendations. The workshops were led by PHu with support from two patient research partners (co-authors, JJ and LR) and recorded on Zoom. Workshops were 2-h duration with a scheduled comfort break. A 4-stage NGT process was followed [12]:

- Stage 1 — initial voting and silent generation
  o Participants were shown up to 10 recommendations at a time and voted on the importance of each recommendation via the Zoom polling function using a 4-point scale (1. Not important/not relevant; 2. Slightly important; 3. Important and 4. Very important).
  o Participants made notes individually of suggestions for additional recommendations (silent generation of ideas).
- Stage 2 — round robin
  o Results from initial voting were shared with the group.
  o In a round robin process participants were invited to add or amend recommendations, ensuring all voices were heard and diverse opinions sought.
- Stage 3 — clarification
  o A discussion on the potential recommendations was facilitated by PHu, JJ and LR. Care was taken to ensure all participants were given an opportunity to contribute.
  o During a short break, polling questions were amended to reflect changes made in the discussion stage.
- Stage 4 — prioritisation by participant ranking
  o Participants were asked to vote on the new list of recommendations created from participant suggestions (intra-group ranking).

Amended and new recommendations were taken forwards from each workshop to be used for the initial polling at the subsequent workshops.

### Data analysis

Participants’ voting scores were transferred to a Microsoft Excel spreadsheet. Voting outcomes were summed across the NGT workshops to derive an inter-group score, following examples in the literature [16, 20–22]. Data were reported as a percentage of the maximum possible score (number of participants × 3 points × 100). This analysis was checked by the PhD supervisory team.

Qualitative data analysis can add valuable meaning and explanation of the quantitative results, optimising participants’ contributions by using all available data [15, 23]. Content analysis (CA) can be used to categorise and count numbers of comments. However, in a group situation, counting or quantifying becomes problematic as it is not possible to determine how many participants agree with a stated view from another participant. Therefore, CA without quantitative data was used. The Zoom recordings were shared with the PhD supervisory team and participant comments summarised by PHu. Inductive CA was carried out by PHu to categorise the verbal data through identification of patterns of meaning and consistencies and checked by the PhD supervisory team. The qualitative data reported represents the thoughts and reflections of PHu through an analytical stance, as an adjunct to the quantitative data captured through voting.

## Results

### Phase 1: Drawing up a list of potential patient care recommendations

Fifty-one potential recommendations were developed (see Table 1).

**Table 1:** A list of potential patient care recommendations

#	Patient care recommendation
1. **Pain management**	
**1.1**	Each centre should have a protocol for anaesthesia for applicator insertion, including options for anaesthesia for different types of applicators and adaptations to meet the needs of individual patients
**1.2**	Each centre should have a protocol for pain management in theatre recovery, including options for pain and relaxant medication for different types of applicators and to meet the needs of individual patients
**1.3**	Each centre should have a protocol for pain management on the ward for the duration with applicators in place, including options for continuous flow or patient controlled pain medication and breakthrough pain to meet needs of individual patients
**1.4**	Each centre should have a protocol for pain management for applicator removal to meet the needs of individual patients
**1.5**	Each centre should provide individualised advice on pain control before discharge from hospital
2. **Medication for anxiety and distress**	
**2.1**	The protocol should include consideration of medication to reduce anxiety while staying on the ward or at home the night before brachytherapy
**2.2**	The protocol should include consideration of patient request or need for drugs to reduce anxiety and distress when coming into theatre
**2.3**	The protocol should include consideration of patient choice or need for drugs to reduce their awareness of the theatre procedure
**2.4**	The protocol should include consideration of patient choice or need for drugs to help patients sleep when on the ward for long duration brachytherapy
**2.5**	The protocol should state the minimum frequency or threshold for pain, anxiety and distress to be reviewed by senior brachytherapy clinicians
**2.6**	The protocol should include frequency of ward rounds with oncologist and nursing staff for regular review and management of pain, anxiety and distress
**2.7**	The protocol should include consideration of patient request or need for drugs to reduce anxiety and distress during applicator removal
**2.8**	The protocol should include consideration of patient choice or need for drugs to reduce their awareness of applicator removal
3. **General medical management**	
**3.1**	Each centre should have a protocol for prevention and treatment of nausea and vomiting, including additional medication options and adaptations when medication doesn’t work
**3.2**	Each centre should have a protocol for prevention of severe infection, including the level of blood count where preventative antibiotics should be given and the level of infection risk with different applicator types
**3.3**	Each centre should have a medical pre-brachytherapy assessment protocol, including when doctors should discuss individual cases to weigh up the risks and benefits of brachytherapy and any adaptations needed
**3.4**	Senior brachytherapy clinicians should consider change of regimen/technique or no brachytherapy if there are significant medical or psychological trauma risks
**3.5**	Each centre should provide a late effects service, to help with possible long term side effects of treatment such as bowel, bladder and sexual problems in the months and years after completion of treatment
**3.6**	Each centre should have a protocol regarding patient positioning and where possible to avoid keeping patients in a totally flat position
**3.7**	Each centre should have a protocol for prevention of blood clots, including risk assessments, how often to re-assess risk and the use of preventative medication and mechanical devices (such as stockings or alternative devices)
**3.8**	Each centre should provide training for brachytherapy clinical staff on pain assessments and understanding individual pain experiences, including the impact of psychological trauma and mental health history, previous pain and analgesia history
**3.9**	Each centre should have a strategy for prevention of pressure sores
4. **Information and support**	
**4.1**	Each centre should allocate appropriate time/resources to patient-centred pre-brachytherapy information and support
**4.2**	Each centre should provide training for the brachytherapy clinical team on potential psychological trauma of cervical cancer diagnosis and triggers for trauma during treatment, especially for brachytherapy
**4.3**	Individual risk assessments to be carried out for potential trauma during brachytherapy, considering factors such as age, social history, previous pain/medication history, mental health, coping mechanisms, and adaptations/access to specialist support
**4.4**	Each centre should provide written and verbal advice at the point of discharge from hospital on management of post treatment side effects and information on accessing help and support
**4.5**	Each centre should provide support to patients after completion of brachytherapy, such as a telephone call a few days after discharge home, offering a debriefing session to talk through what happened and offering advice on management of aftereffects
**4.6**	Each centre should provide information about patient support groups that the individual can access after completion of cancer treatment
**4.7**	Each centre should provide assessment of the need for psychological support after brachytherapy
5. **Patient care/ward nursing care**	
**5.1**	Ward nurses should offer advice and support in relation to eating and drinking while applicators are in place
**5.2**	Ward nurses should receive training about nutrition requirements and the need to monitor patients during brachytherapy to ensure they are supported to eat
**5.3**	Wards should provide access to someone for the patient to communicate with when lying flat with applicators in place, especially if visiting is restricted
**5.4**	Ward nurses should check in on patients at regular frequent intervals and provide support through the night if patients are unable to sleep due to pain/discomfort/distress
**5.5**	Ward nurses should offer help and support with personal care
**5.6**	Ward nurses should provide close supervision of patients after applicator removal to avoid risk of falls and monitor the effect of medication wearing off
**5.7**	Ward nurses should help patients to prepare for discharge home, including washing, dressing and mobilising
**5.8**	Ward staff should receive training on awareness and identification of drug reactions, especially for long duration brachytherapy or high levels of opiate use
**5.9**	Ward staff should receive training in care and compassion, understanding the patient journey though the cancer diagnosis and treatment, including brachytherapy
**5.10**	Centres should provide intensified care standards for brachytherapy patients on ward, ie fewer patients that one nurse should be allocated to look after, therefore a greater allocation of nursing time to brachytherapy patients
6. **Communication, logistics and staffing**	
**6.1**	Each centre should ensure that there is effective communication between referring centres and brachytherapy teams, especially where plans change including dates for treatment or centre for brachytherapy
**6.2**	Each centre should offer transport for patients to attend brachytherapy and return home after brachytherapy, if there are no family/friends able to provide
**6.3**	Each centre should carry out regular service evaluation to check that staffing levels are appropriate throughout the brachytherapy pathway
**6.4**	Each centre should implement a service evaluation programme for obtaining patient feedback about their brachytherapy services, including patient reported pain and distress, especially after adaptations to service delivery are made or new services introduced
**6.5**	Each centre should ensure that patients do not experience delays to treatment or unnecessary transfers
7. **Facilities on wards**	
**7.1**	Centres should where possible offer patients a choice of a single room or shared ward room, considering individual preferences for privacy or company/distractions
**7.2**	Centres should provide clear information to patients about access to facilities such as TV, internet and music to help pass the time
**7.3**	Centres should provide access to facilities such as an angled tray for reading and/or iPad to optimise patient comfort and enable access to facilities when lying flat for a long period of time
**7.4**	Centres should offer complementary therapies during admission for brachytherapy
**7.5**	Centres should provide information and support to help patient’s use of relaxation techniques during admission for brachytherapy
**7.6**	Centres should provide pre-brachytherapy information to patients including detail of ward facilities, what to bring in, what to expect and to offer to show patients around in advance of brachytherapy
**7.7**	Centres should offer patients a choice of brachytherapy regimen, where possible and equally effective

### Phase 2: Nominal group technique (NGT) workshops

Email enquiries were received from 46 potential participants, with 10 service users and 22 service providers subsequently completing the Qualtrics eligibility survey and consent form. Scheduling workshops was problematic due to availability. Three NGT workshops took place between March and April 2022, with a total of 13 participants taking part. Numbers of service providers and service users per workshop are shown in Table 2. The service providers were from 3 different healthcare professions: 1 clinical oncologist; 3 nurses and 4 radiographers. Overall, participants had experience of brachytherapy at 9 UK brachytherapy centres with a mixture of experiences of short duration (day case) and long duration brachytherapy (at least 1 overnight stay with applicators in place). Workshop participants were from brachytherapy centres in England and Northern Ireland.

**Table 2:** Workshop service user and service provider participant numbers

	Workshop 1	Workshop 2	Workshop 3	Total
Number of service users	2	2	1	5
Number of service providers	3	2	3	8
Total number of participants	5	4	4	13

### Polling

Workshop 1 began with participants rating the 51 potential patient care recommendations. Participants were invited to suggest changes to wording of recommendations or additional recommendations they would like considered and to discuss this within the group. Four additional recommendations were agreed by the participants. The Zoom screen share function was used to display and edit the recommendation list, reflecting the group decisions on changes and additions required. Wording amendments or further text for clarification were added to 15 recommendations. The new list of 55 recommendations was used for the 2nd poll at workshop 1 and for initial polling at workshop 2. At workshop 2, wording was amended to 1 recommendation, and no new recommendations were developed. This amended list was used for the 2nd poll at workshop 2 and initial polling at workshop 3. Wording amendments or text for clarification for 3 recommendations were agreed at workshop 3 and taken forward for the 2nd poll (see Table 3 for new and amended recommendations).

**Table 3:** New and amended recommendations

Workshop 1: new recommendations		
Poll number and title	**New recommendation**	**Comments**
Poll 4: information and support	*4.8 Pain management, methods, and potential side effects should be discussed with patients before, during, and after treatment, with level of detail and choices offered as appropriate*	Suggested by a service user who had not had pain management and opiate side effects explained to her and reported suffering from paranoid thoughts likely to have been caused by high levels of opioid use via PCA
Poll 6: communication, logistics and staffing	*6.6 Ward bookings for long duration brachytherapy should include the option to stay the night after treatment finishes, to allow sufficient recovery time if needed*	Suggested by a service user who had been encouraged to leave hospital when she did not feel ready. She suggested that patients should be given a choice, to stay on ward for an extra night after brachytherapy completion
Poll 6: communication, logistics and staffing	*6.7 Pregnancy checks before theatre procedures and radiation delivery should be handled with sensitivity where previous treatment has prevented this possibility*	Suggested by a service user who had found the frequent questions about the possibility of pregnancy was upsetting and insensitive when treatment had caused infertility
Poll 7: facilities on wards	*7.8 Consideration should be given to the location of brachytherapy ward facilities and where possible avoid entry and exit routes near sensitive areas such as maternity units*	Suggested by a service user who had to walk through a maternity unit to reach the ward for brachytherapy. She found this upsetting, insensitive and hoped it might be considered when designing future services
Workshop 1: recommendation amendments		
Poll number, title, recommendation number	**Original recommendation**	**Amended recommendation**
Poll 1: pain management. Recommendation 5	Each centre should provide individualised advice on pain control before discharge from hospital	Each centre should provide individualised advice on *short term pain management* before discharge from hospital
Poll 2: medication for anxiety and distress. Recommendation 1	The protocol should include consideration of medication to reduce anxiety while staying on the ward the night before brachytherapy	The protocol should include consideration of medication or other interventions to reduce anxiety while staying on the ward *or at home* the night before brachytherapy
Poll 2: medication for anxiety and distress. Recommendation 5	The protocol should state the frequency that pain, anxiety and distress will be reviewed by senior brachytherapy clinicians	The protocol should state the *minimum* frequency that pain, anxiety and distress will be reviewed by senior brachytherapy clinicians
Poll 2: management of anxiety and distress. Recommendation 6	The protocol should include frequency of ward rounds with oncologist and nursing staff for regular review and management of pain, anxiety and distress	… and management of pain, anxiety and distress, *in addition to personalised reviews at times needed by the patient*
Poll 2: Title: medication for anxiety and distress	Poll title: medication for anxiety and distress	Poll title: *Management of anxiety and distress*
Poll 2: medication for anxiety and distress. Recommendations 1, 2 3, 4, 7 and 8	…need for drugs…	Six recommendations had *“or other interventions”* added, to read: need for drugs *or other interventions*
Poll 3: general medical management. Recommendation 1	Each centre should have a protocol for prevention and treatment of nausea and vomiting, including additional options and adaptations when medication does not work	…when medication does not work, and consideration of non-medical options*. Explanatory notes: non-medical options may include herbal remedies such as ginger, relaxation techniques, music therapy or massage*
Poll number, title, recommendation number	**Original recommendation**	**Amended recommendation**
Poll 4: information and support. Recommendation 7	Each centre should provide assessment of the need for psychological support after brachytherapy	Each centre should provide assessment of the need for psychological support after brachytherapy and *be able to provide this or refer patients as needed*
Poll 5: patient care/ward nursing care. Recommendation 9	Ward staff should receive additional training in the care and compassion needed to support patients during brachytherapy	*Explanatory notes: This may include training such as advanced communication skills, a need for clinical supervision sessions or staff debriefing*
Poll 6: communication, logistics and staffing. Recommendation 3	Each centre should carry out regular service evaluation to check that staffing levels are appropriate throughout the brachytherapy pathway	Each centre should carry out regular service evaluation to check that staffing levels are appropriate throughout the brachytherapy pathway*, including contingency planning for absence of key staff*
Poll 7: facilities on wards. Recommendation 1	Centres should offer patients a choice of single room or wardroom, considering individual preferences for privacy or company/distractions	Centres should *where possible* offer patients a choice of a single room or *shared* wardroom, considering individual preferences for privacy or company/distractions
Workshop 2: Recommendation amendments		
Poll 5: patient care/ward nursing care. Recommendation 10	Centres should provide intensified care standards for brachytherapy patients on ward, i.e. fewer patients that one nurse should be allocated to look after, therefore a greater allocation of nursing time to brachytherapy patients	Centres should provide *specialised* care standards for brachytherapy patients on ward, i.e. fewer patients that one nurse should be allocated to look after, therefore a greater allocation of nursing time to brachytherapy patients
Workshop 3: Recommendation amendments		
Poll 1: pain management. Recommendation 4	Each centre should have a protocol for pain management for applicator removal to meet the needs of individual patients	Each centre should have a protocol for pain management for applicator removal to meet the needs of individual patients *(fully informed of procedure)*
Poll 2: management of anxiety and distress. Recommendation 5	The protocol should state the minimum frequency that pain, anxiety and distress will be reviewed by senior brachytherapy clinicians	The protocol should state the minimum frequency *or threshold* for pain, anxiety and distress to be reviewed by senior brachytherapy clinicians or senior ward clinicians
Poll 5: patient care/ward nursing care. Recommendation 9	Ward staff should receive additional training in the care and compassion needed to support patients during brachytherapy *This may include training such as advanced communication skills and a need for clinical supervision sessions or debriefing for staff*	Ward staff should receive additional training in the *nursing care* and compassion needed to support patients during brachytherapy *This may include training such as advanced communication skills and a need for clinical supervision sessions or debriefing for staff, and management of complex pain*

Three points were allocated for “very important” responses; two points for “important”; one point for “somewhat important” and no points for “not important/not relevant”. Polling results from the 2nd poll at each workshop were summed and calculated as a percentage of the maximum score possible. Over the 3 workshops, 25 recommendations received a score of 100%, the maximum possible score, showing that all participants across the three workshops voted “very important” for these 25 recommendations. Overall, 46 recommendations received a score of 90% or above, and nine recommendations received a score of less than 90% with a lowest score of 74%. When votes from 2nd polls across the 3 workshops were grouped by participant type, service users versus service providers, there was no significant difference seen in voting patterns. When votes from second polls across the 3 workshops were grouped by brachytherapy type, short duration experience versus long duration experience, there was no significant difference seen in voting patterns. Of the 9 recommendations which received overall scores lower than 90%, 6 of these were in poll 7, relating to facilities onwards. For example, offering a choice of a single or shared wardroom, complementary or relaxation techniques and facilities to help women pass the time while lying flat for long periods of time. For 2nd poll results, see Table 4.

**Table 4.**
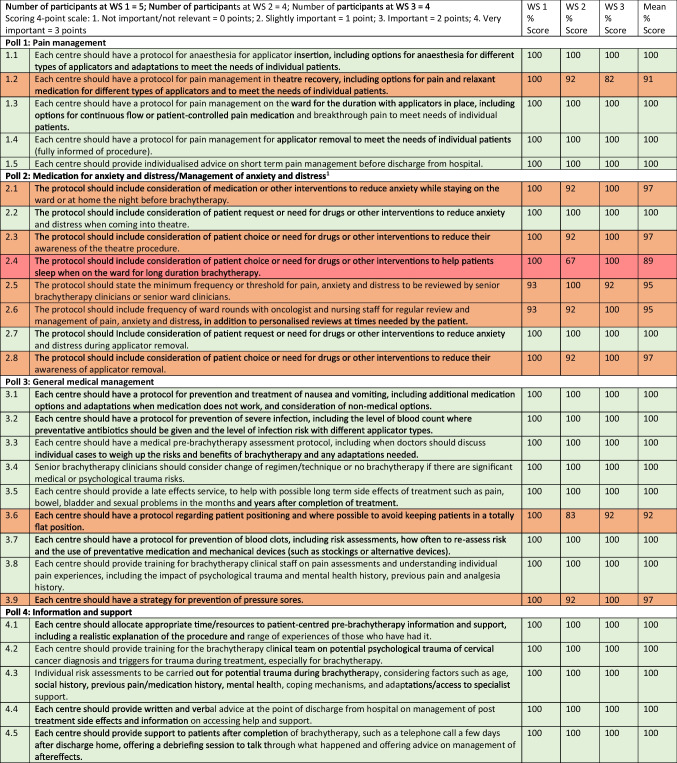

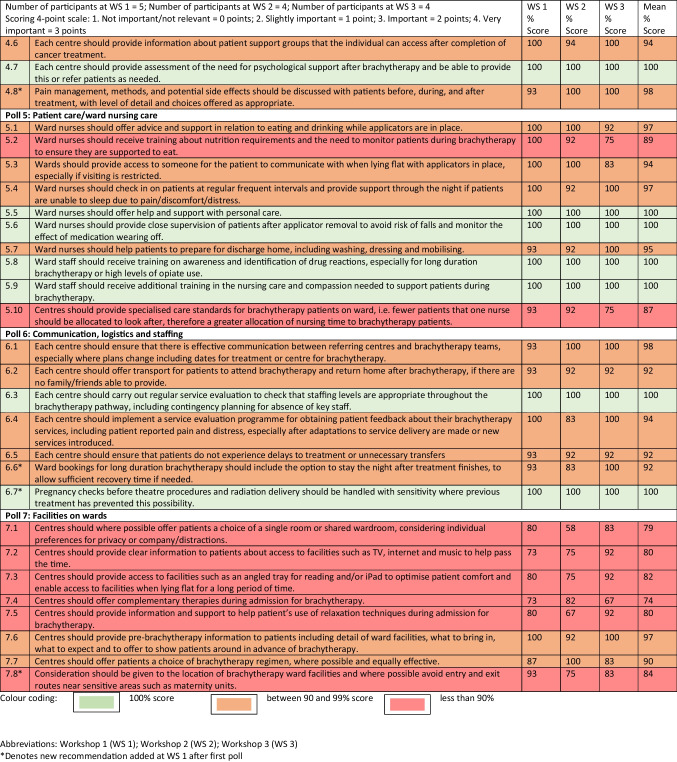
NGT Workshop polling results (2nd poll)

In the ‘Round robin’ stage, participants were asked in turn to comment on their reasons or justification for their choice of ratings in the initial voting round. Many participants commented on how important all the recommendations were and that ideally, they would all be included in future recommendations.

### Content analysis of qualitative data from stage two and three

Using a content analysis method, verbal data from all workshops were grouped into themes and themes organised into overarching categories, as reported in Table 5.

**Table 5:** Content analysis of qualitative data from NGT workshops

**Round robin individual comments and discussion of proposed patient care recommendations for brachytherapy at 3 workshops** **Total number of participants = 13 (service users = 5; service providers = **8)	
**Category**	Examples of verbal responses
**Rationale for scoring**	All proposed recommendations are important
	Pain management, information and psychological support are most important
	Some recommendations are not required as they should be standard care
	Some recommendations are not necessary as already in place
	Feasible recommendations are most important
	Feasibility should not be the main driver of prioritisation
	Complementary therapies are relatively less important
**Pain**	Patients have fear of pain before brachy
	Inadequate pain management was experienced
	Some experienced or provided good pain management
	Severe pain significantly impacts on patient experience
**Patient information and support**	Patient centred, timely and accurate patient information is very important
	Inaccurate or unrealistic information was reported
	Patient information about aftereffects and aftercare is very important
	Patient information about ward facilities is helpful
	Patient information before discharge home is very important
	Distress was caused by poor timing of brachy information
	Poor patient information impacts patient experience
**Ward care and training**	Inconsistent and poor care on wards was reported
	Some good nursing care was reported
	Brachytherapy training of ward nurses/HCPs needs improvement
	Guidelines for ward brachy nursing care are needed
	Inconsistent staff allocation was reported
	Nutrition and hydration support need improvement
	HCPs need training in complex pain
	Need to help patients to access time passing strategies whilst lying flat
	Guidelines to minimise pressure sores and DVTs are needed
	Ward nurses need advanced communication skills training
	Need to improve ward medication administration
	Brachy trained ward staff gives more consistent good care
	Lack of training can lead to poor patient experiences and complaints
**Facilities**	Inappropriate ward location for brachytherapy patients
	Providing single rooms is not always feasible
	Single bay inpatient bed are available in some centres
	Appropriate logistical resources are needed, such as individualised transport
	Should offer patient choice for length of inpatient stay
**Physical effects of brachytherapy**	Excess side effects from medication
	Offer pharmacological and non-pharmacological for nausea and vomiting
	Flat position for brachy is problematic
	Total reliance on help from partner/relative to eat and drink
	Poor management of side effects was reported
**Late effects**	Late effects service provision is really important
	Experience of late effects reported and poor information and support
**Psychological issues**	Need to increase sensitivity and awareness of HCPs to fertility loss and impact
	Emotional or psychological impact of brachytherapy was reported
	Psychology expert advice is needed for debriefing recommendation
	Management of anxiety needs improvement
	Patients experience stress and anxiety before, during and after brachy
	Report of feeling vulnerable and disempowered due to flat position
**Short duration versus long duration brachytherapy**	Good care was experienced for short duration brachy
	Increased duration can be for complex reasons, hard for patients to tolerate
	Different views on duration of brachytherapy
	Day case brachy gives better experiences than inpatient (long duration)
	Would have preferred day case brachytherapy
	Preferred all brachytherapy in one admission
**Anaesthetics**	Lack of consistency of care in anaesthesia reported
	Having experienced anaesthetists is important
**Patient-centred care**	HCPs need to know patients well deliver patient-centred care
	Building rapport helps to identify patients with higher risk of trauma
**Miscellaneous**	Care delivered differently in centres
	Patient feedback is used to improve their next brachy experience
	Patient experienced good support and care in theatre
	Staff work hard to try to make patient experiences tolerable
	Good communication between centres is essential
	Need to improve logistics for pre-assessment
	Radiographers provide ward nursing care too
	Some patient experiences are good
	Patient choice is important
	Patient comfort needs to be improved
	Privacy versus companionship was discussed

*HCPs* healthcare professionals, *DVT* deep vein thrombosis, *brachy* brachytherapy

### Reflections on qualitative data and examples for illustration

Overall, service users and providers were very supportive of the list of proposed recommendations. One service provider said:

“Every single step is really important as we need to get every step right” (Service provider, workshop 1)

This was corroborated by a service user who commented:

“All of it I kind of think is really important, and it will be brilliant to see this, and it will make such a massive difference if this is done, for the consistency across treatment centres.” (Service user, workshop 1)

Some discrepancies were observed between experiences of service user and service provider participants. Overall, service users were more negative than service providers, especially regarding consistency of nursing care, management of pain and other physical side effects. They spoke of experiencing some aspects of good care, but also that care was inconsistent and, at times, poor. Three service users had experienced poor pain management with long duration brachytherapy, with one participant experiencing flashbacks of applicator removal, describing it as “barbaric” and “cruel”. In contrast, some service providers said that brachytherapy ward staffs were trained and that good care and provision of good pain management was standard practice.

The service users highlighted the importance of recommendations that resonated with negative experiences of brachytherapy, such as problems with pain, nausea and vomiting, lying flat, reaching food and drinks, feeling wobbly after applicator removal, and that their voting was an affirmation of the importance of improving these negative experiences. In contrast, some service providers voted more strongly in favour of recommendations that would be feasible or more important in their centres. A service provider in workshop 1 said that all recommendations were important but that in relative terms, patient preparation through information or pain management “trumps” services such as complementary therapies. Other service providers said that some proposed recommendations were not required or less relevant in their centre, as those aspects were already in place. Some service providers expressed concerns about resources and that some recommendations were aspirational but not currently possible.

Service users emphasised the importance of the recommendation relating to the provision of ‘late effects’ services. One service user thought the information leaflets provided by hospitals gave “false information” about late effects of treatment and had lost faith in NHS information websites. In contrast, service providers did not comment on late effects services or the proposed recommendation; however this recommendation received 100% scores for importance at all workshops.

Some service providers expressed their appreciation for the participation of service users at the workshop, emphasising the importance and impact of hearing patient voices. One service provider participant provided feedback via email after the workshop:

“I thoroughly enjoyed the workshop and found it very informative to hear what other clinics are doing and the patients’ perspective was particularly valuable.”

## Discussion

Three online NGT workshops were carried out with 5 service users and 8 service providers. Overall, recommendations were positively received, and some new recommendations developed.

Service users commented on the value of the proposed patient care recommendations due to their resonance with problems they had experienced during brachytherapy and a desire to see the recommendations implemented in clinical practice. They placed particular importance on the need for pain management at key time points. National guidelines for management of acute pain include use of individualised analgesia plans which are safe, effective and regularly reviewed [24]. However, a lack of consistency in approach to acute pain management and highly variable service provision has previously been found [25]. This finding was verified by service users in the NGT workshops.

Morris and Haboubi [26] state that pelvic radiation disease has been under reported and sub-optimally treated over many years, with an over emphasis on survival rates and a neglect of short- and long-term toxicity of treatment. UK service specifications for radiotherapy include requirements for provision of specialist late effects centres [27]. In this study, the need for late effects services was regarded by workshop service users to be particularly important. Some had personal experience of radiotherapy late effects and were keen to see a higher prioritisation of improving this service provision.

Two of the new recommendations are related to patient choice. Firstly, recommending that full information about potential side effects of pain medication should be given so that informed choices could be made by patients. Medication prescribing recommendations in the UK state that healthcare professionals should advise patients on adverse effects and potential harm and involve them in shared decision making, including realistic expectations, to enable informed decisions [24]. For brachytherapy, this information provision and discussion would ideally take place in the pre-operative assessment setting to inform patients about potential pain and likely analgesia requirements. Secondly, patients should be given a choice about whether to stay the night after brachytherapy. With the limited availability of ward beds, some service providers were not sure if this would be possible in their centres.

The other new recommendations related to the need to improve sensitivity about infertility are caused by cancer treatment. Being asked multiple times during radiotherapy and theatre procedures if there was any possibility of pregnancy and needing to walk through maternity areas to access radiotherapy or brachytherapy services was reported to have caused distress. Although service providers commented that relocating facilities may be difficult, all participants agreed that this should be considered in future service design.

Recommendations in Poll 7 (Facilities on wards) received the lowest scores overall and included 6 of the 9 recommendations with less than 90% scores. Both service users and service providers discussed the difficulties of resource allocation, such as access to complementary therapies, support for relaxation techniques and facilities to help women cope with lying flat for prolonged periods of time. Some participants believed that offering a choice of a single room or a shared wardroom was unrealistic due to the limited availability of beds. However, some service providers were keen for inclusion of aspirational recommendations to support subsequent requests for better resource allocation. Using patient experience feedback to drive service improvements has long been recommended [28, 29], putting patients at the centre of developments. It is recognised that changes based upon patient experience feedback need to be balanced against clinical effectiveness and safety improvements. However, integration of patient feedback with staff experiences has been demonstrated though initiatives such as ‘Experience Based Co-Design’, driving improvements that are relevant and feasible [30].

Reflecting the importance of co-production in this study, 2 patient research partners were involved in designing the workshops, reviewing documents and participation in the NGT workshops as co-facilitators. The co-design method, bringing service users and providers together to discuss and share their experiences, was recognised by participants as having impact and adding value to the workshops. This is considered a worthwhile ambition and strength of the study, despite the difficulties in scheduling workshops. The NGT online format worked well, facilitating fairness and equity between service users and service provider participants, through equal allocation of time and the use of polling.

The number of participants was lower than originally planned and could be seen as a limitation of this study, as larger participant numbers could have included a broader range of views, experiences and knowledge from both service users and service providers. However, the small number of participants enabled detailed discussion of the 55 proposed recommendations as there was more time for each participant to explain their views.

Overall, the aims and objectives of the study were met as service users and providers were able to discuss, refine and prioritise the potential patient care recommendations, leading to the verification of useful, potentially achievable and relevant recommendations.

## Conclusions

Proposed patient care recommendations were discussed and voted on at 3 workshops that included both service user and service provider participants. Of the 55 potential recommendations, all participants considered 25 recommendations to be very important, receiving the maximum possible score of 100%. Scores of 90% or above were given to 46 of the 55 recommendations. Verbal comments from participants confirmed that all the recommendations were relevant and important. The 55 patient care recommendations provide a foundation for piloting an implementation programme in a small number of centres, to lead on to a wider roll out into clinical practice. Further work is planned to develop patient care guidelines through collaboration with national professional bodies and relevant international organisations. The aim of the guidance would be to improve consistency of care and patient experiences of brachytherapy across centres with different brachytherapy techniques and regimens.

The views expressed in this publication are those of the authors and not necessarily those of the NIHR, UWE Bristol, NHS or the UK Department of Health and Social Care.

## Data Availability

The datasets generated and analysed during the current study are not publicly available due to containing information that could compromise the privacy of research participants.

## Declarations

The views expressed in this publication are those of the authors and not necessarily those of the NIHR, UWE Bristol, NHS or the UK Department of Health and Social Care.
